# Healthcare-Associated Infections: Pre- and Post-pandemic Microbiological Profile and Antibiotic Resistance

**DOI:** 10.7759/cureus.70263

**Published:** 2024-09-26

**Authors:** Erick Sierra-Diaz, Gabriel Puron-Cid, Joselyne Paola Torres-Sanchez, Héctor Israel García-Quintero, Diana Lorena Cisneros-García, Mariana García-Gutierrez, Rosa Cremades, Elena Sandoval-Pinto

**Affiliations:** 1 Epidemiology and Public Health, Mexican Social Security Institute, Guadalajara, MEX; 2 Public Administration, Center for Economic Research and Teaching, A.C., Aguascalientes, MEX; 3 Epidemiology, Mexican Social Security Institute, Guadalajara, MEX; 4 Public Health, University of Guadalajara, Guadalajara, MEX; 5 Pediatrics, School of Medicine and Health Sciences, Monterrey Institute of Technology and Higher Education, Guadalajara, MEX; 6 Microbiology and Pathology, University of Guadalajara, Guadalajara, MEX; 7 Cell and Molecular Biology, University of Guadalajara, Guadalajara, MEX

**Keywords:** antibiotic resistance, control, healthcare-associated infections, microbiological profile, notification, prevention, sars-cov-2

## Abstract

Background: In recent years, healthcare-associated infections (HAIs) have been a priority topic for global institutions such as the World Health Organization because, during the COVID-19 pandemic, the role of HAIs as co-morbidity in infected patients was evident. HAIs can cause disability and mortality and lead to excessive healthcare costs. This work aims to calculate the prevalence of HAIs from 2019 to 2023 to determine their microbiological profile and antibiotic resistance.

Methods: A cross-sectional design was used for this study. To describe the population, univariate analysis, measures of central tendency, frequencies, and proportions were used.

Results: The present study included 3,936 HAI notifications, which showed a significant decrease in their number during the COVID-19 pandemic, especially in 2020. In 2021 and 2022, the numbers increased rapidly to around 30%. The most prevalent HAI type was ventilator-associated pneumonia, which had the highest prevalence in 2020. Regarding microorganism isolation, percentages increased after 2020 from 46% to 67%, with *Acinetobacter baumannnii *beingthe most frequent during and after pandemics. The microbiological profile showed multidrug resistance in several microorganisms.

Conclusions: HAIs are a global health concern. The number of cases has been increasing since the COVID-19 pandemic with regard to the multidrug-resistant microorganism. HAIs have an important impact on the quality of life, morbidity, mortality, and financial concerns for health services. Global strategies should be adapted for different regions, since the panaroma in developed countries allows for programs to be established for the prevention and control of HAIs in a better way than in low-income countries that lack adequate infrastructure and resources.

## Introduction

Healthcare-associated infections (HAIs) are a global public health problem. HAIs are reported as being one of the most frequent complications for patients when entering a health service facility. The World Health Organization (WHO) defines them as “an infection occurring in a patient during the process of care in a hospital or other health care facility that was not present or incubating at the time of admission. HAIs can affect patients in any type of setting where they receive care and can also appear after discharge” [1].

HAIs not only increase hospital stay but also generate long-term sequelae, resulting in increased costs in health care services, mortality, and a decrease in the family quality of life. Other types of effects involve an impairment of economic development, public administration, the efficiency of utilizing resources, and the quality of service provision [1,2].

In Mexico, there is federal regulation that is based on the Official Mexican Standards, whose purpose is to establish parameters and indicators for measurement. At the federal level, epidemiological surveillance (ES) of HAIs is carried out through the Hospital Epidemiological Surveillance Network (RHOVE), which integrates data from hospitals in the health sector as well as from private sector health institutions that are registered in the network [2]. The Mexican Social Security Institute (IMSS) has a system that is not integrated into RHOVE. This system is called INOSO (Nosocomial Infections, IMSS registration platform). It is an institutional platform that feeds ES to the IMSS units [3]. INOSO concentrates the frequency of infections by HAIs type, as well as the microbiological profile of each of the IMSS units. The feeding of information into INOSO depends on two fundamental factors that are based on the Official Mexican Standard PROY-NOM-045-SSA-2024 for Epidemiological Surveillance, Prevention, and Control of HAIs [4]. The first involves timely notification by health personnel who are in direct contact with the patient (attending physician, residents, nursing, etc.). The second involves active surveillance conducted by Hospital Epidemiological Surveillance Units (ESU).

The WHO reported that there are approximately 100,000 cases of mortality in the United States per year. In Europe, reports indicate about 37,000 deaths annually from HAIs. An important fact that should be highlighted is that mortality from HAI doubles when there is resistance to antibiotics [5]. Regarding the type of infection, in the United States, the distribution of mortality due to HAIs is the following: ventilator-associated pneumonia (VAP) (14.4%), catheter-related bloodstream infection (CRBI) (12.3%), surgical site infection (SSI) (2.8%), and urinary catheter-associated urinary tract infection (UTI) (2.3%) [6]. A study published in 2013 estimated that the cost of managing HAIs in the United States is $10 billion per year [7]. In countries where a specific metric of the distribution of HAIs is used, VAP is ranked first, followed by CVC-STIs [6]. However, other authors report that the HAIs that generate the most expenditure are SSIs [8].

As mentioned previously, the causes and behavior of the epidemiology of HAIs can be diverse. The objective of this work is to determine the behavior of HAIs in 2019 as a base frequency and to compare it with 2020, which was the beginning of the pandemic, and 2021 when mortality peaked during the pandemic in the unit, coupled with the variable of the introduction of vaccines. The frequencies from 2019 to 2021 were compared with the frequencies of “the new normal” in 2022. From the results obtained, it showed a significant decrease in cases near the end of the pandemic, which was declared by the WHO in 2023.

## Materials and methods

A cross-sectional study was carried out in the Division of Epidemiology at the Western National Medical Center, Guadalajara, Mexico, using the databases of the same Division, among which is INOSO, which is the official platform of IMSS. The study period covered January 2019 to August 2023.

The data collected was divided by epidemiological year and the variables (type of infection, isolated microorganisms, resistance to antibiotics, and type of notification) and were filtered to obtain the results set out in the objectives. The above was granted with prior consent from local Ethics and Research Committees, with registration R-2023-1301-189/COFEPRIS 17 CI 14 039 114/COMBIOETICA 14 CEI 30290123.

HAIs types and indicators

There are different types of HAIs that can affect different organs and systems. In IMSS, the calculation of six indicators is stipulated in the Methodological Manual [9]. These indicators include the four most important types of HAIs for IMSS, which are reported in INOSO monthly, and feedback is received regarding the frequency of each. The HAIs that measure the indicators are SSI, VAP, CRBI, and last, UTI. These correspond to the IN_AAS 02-05 indicators, respectively. The HAIs indicators IN_AAS 01 and 06 correspond to the total HAIs in the unit per time and the total HAIs in critical care units, respectively.

Data were organized into spreadsheets, and univariate analyses were used to describe the study population. The prevalence rate was calculated with conventional methods using Excel V16.7® (Microsoft Corporation, Redmont, USA), and for the analysis of the resistance of the isolated microorganisms, the automated Vitek® System (bioMérieux, Marcy-l'Étoile, France).

## Results

A total of 3,936 patients with an HAIs diagnosis were included in the 54-month period. Of the population studied, 60.5% (n=2355) were men, and the average age of the group was 53.5 years. Table 1 shows the details regarding gender and age by year of study.

**Table 1:** Demographic data of patients with positive cultures by year SD: standard deviation

Demographic data of patients with positive cultures per year																		
Year	2019			2020			2021			2022			2023			Whole period		
Gender	n (%)	Age mean	SD	n (%)	Age mean	SD	n (%)	Age mean	SD	n (%)	Age mean	SD	n (%)	Age mean	SD	N (%)	Age mean	
Female	392 (39%)	54.20	3.42	232 (36%)	56.60	2.453	406 (44%)	55.27	3.67	380 (40%)	53.42	3.86	171 (43%)	52.70	1.69	1581 (40%)		54.46
Male	623 (61%)	54.64	4.88	420 (53%)	53.1	3.736	513 (56%)	51.7	4.59	571 (60%)	53.75	4.38	228 (57%)	50.242	2.45	2355 (60%)		52.68
Overall	1015 (100%)	55.54	6.76	652 (100%)	55.18	4.90	919 (100%)	54.11	6.69	951 (100%)	53.58	6.03	399 (100%)	51.24	2.98	3936 (100%)		53.57

The prevalence of HAIs in the period was 46.33 per 1,000 discharges. Table 2 shows the prevalence of HAIs for each year of study.

**Table 2:** Prevalence of HAIs per year ^* ^indicates the first eight months of 2023 HAIs: healthcare-associated infections

Year	Total	Prevalence (per 1000 discharges)
2019	1015	47.97
2020	652	46.53
2021	919	53.83
2022	951	48.64
2023^*^	399	30.32
Total	3936	46.33

**Figure 1:**
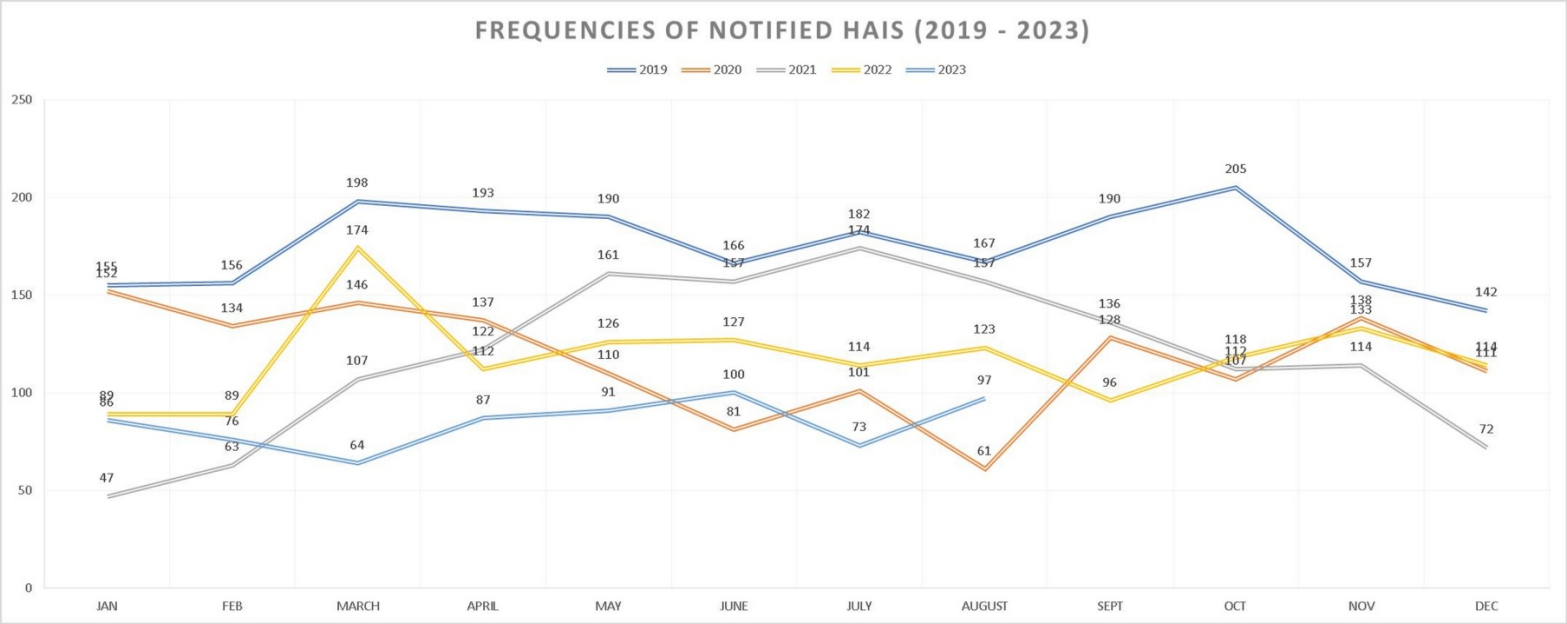
Frequency of HAIs through 54 months of study HAIs: healthcare-associated infections

During the study period, the number of HAIs per month was quantified, noting year-to-year variability. Figure 1 shows the number of cases per month during the 54 months of the study.

**Figure 2:**
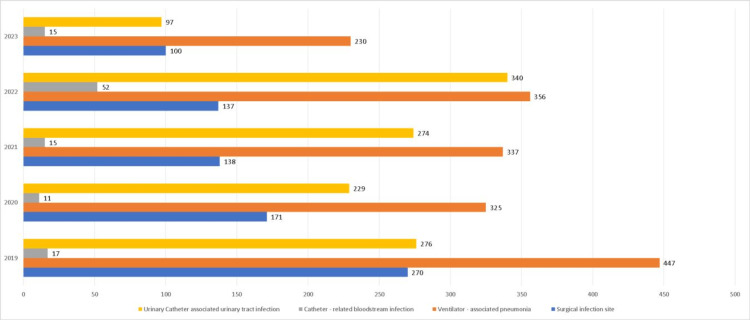
Frequency of the main HAIs per year HAIs: healthcare-associated infections

In 2019, the number of HAIs was practically double that which was cumulative in 2020. The above is directly related to the conversion of the hospital due to COVID-19. During 2023, the frequency of HAIs is lower since, after the pandemic, the use of infection control and prevention packages was intensified [10-12]. Figure 2 shows the frequency of the four main HAIs by years of study. Table 3 complements the frequency with calculations of the prevalence of each of the HAIs.

**Table 3:** Prevalence rate by year SSI: surgical site infection; VAP: ventilator-associated pneumonia; CRBI: catheter-related bloodstream infection; UTI: urinary catheter associated urinary tract infection (per 1000 discharges)

Year	Discharges	SSI	VAP	CRBI	UTI
2019	21160	12.76	21.12	0.80	13.04
2020	14013	12.20	23.19	0.78	16.34
2021	17071	8.08	19.74	0.88	16.05
2022	19551	7.01	18.21	2.66	17.39
2023	13161	7.60	17.48	1.14	7.37

Regarding the cultures performed on patients with suspected HAIs, a total of 7,018 cultures were performed. In 57% of the cultures (3,936) they were positive, with the isolation of various microorganisms. Table 4 shows the percentage of positive cultures per year.

**Table 4:** Frequency of microorganism isolated in cultures per year

	2019	2020	2021	2022	2023
Positive cultures	1015	652	919	951	399
%	48.31%	46.37%	64.63%	67.21%	59.20%
Total	2101	1406	1422	1415	674

The data in Table 4 shows that the positivity of the cultures (<50%) did not present significant changes between 2019 and 2020. However, it should be noted that the number of cultures for 2020 decreased by close to 50%, and after that decrease, an increase of 41% was observed for 2021 and 46% for 2022, respectively. For 2023, the trend observed after eight months suggests lower frequencies than the previous year, with a percent positivity of eight points below.

Among the microorganisms isolated during the study period, *Escherichia coli *and *Acinetobacter baumannii* were placed at the top of the list. The first was in 2019, and the second from 2020 to 2023. Table 5 shows the frequency of the five main microorganisms isolated per year.

**Table 5:** Frequencies and percentages of isolated microorganisms per year

Frequencies and percentages of isolated microorganisms per year											
Isolated microorganism	2019 (n=1015)	Isolated microorganism	2020 (n=652)	Isolated microorganism	2021 (n=919)	Isolated microorganism	2022 (n=951)	Isolated microorganism	2023 (n=399)		
	n %		n %		n %		n %		n %		
										Escherichia coli	197 (19%)	Acinetobacter baumannii	116 (18%)	Acinetobacter baumannii	183 (20%)	Acinetobacter baumannii	153 (16%)	Acinetobacter baumannii	80 (20%)		
Acinetobacter baumannii	173 (17%)	Escherichia coli	99 (15%)	Escherichia coli	140 (15%)	Escherichia coli	141 (15%)	Escherichia coli	52 (13%)		
Pseudomonas aeruginosa	125 (12%)	Pseudomonas aeruginosa	81 (12%)	Pseudomonas aeruginosa	120 (13%)	Pseudomonas aeruginosa	124 (13%)	Pseudomonas aeruginosa	42 (11%)		
Klebsiella pneumoniae	91 (9%)	Klebsiella pneumoniae	52 (8%)	Klebsiella pneumoniae	80 (9%)	Klebsiella pneumoniae	71 (8%)	Staphylococcus aureus	36 (9%)		
Staphylococcus aureus	80 (8%)	Staphylococcus aureus	52 (8%)	Staphylococcus aureus	53 (6%)	Staphylococcus aureus	63 (7%)	Staphylococcus epidermidis	30 (8%)		

According to the institutional indicators that are based on the four most frequent types of HAIs, the frequency of microorganisms by the type of infection was analyzed in each year of study. The information in Table 6 shows a detail of the isolated microorganisms and percentages in each type of HAI.

**Table 6:** Institutional HAI indicator (microbiological profile per year) HAI: healthcare-associated infection

YEAR	Surgical site Infection		%		Ventilator-associated pneumonia			%	Catheter-related bloodstream infection		%	Urinary Catheter associated urinary tract infection			%
2019	Escherichia coli	56		20.74%		Acinetobacter baumannii	61	14.65%	Klebsiella pneumoniae	2	11.76%		Escherichia coli	38	13.77%
	Acinetobacter baumanni	i23		8.52%		Klebsiella pneumoniae	30	6.71%	Pseudomonas aeruginosa	2	11.76%		Pseudomonas aeruginosa	25	9.06%
	Staphylococcus aureus	19		7.04%		Staphylococcus aureus	30	6.71%	Staphylococcus epidermidis	2	11.76%		Candida albicans	12	4.35%
	Klebsiella pneumoniae	13		4.81%		Pseudomonas aeruginosa	24	5.37%	Candida albicans	1	5.88%		Candida Tropicales	12	4.35%
	Pseudomonas aeruginosa	11		4.07%		Candida albicans	15	3.36%	Candida parapsilosis	1	5.88%		Acinetobacter baumannii	7	2.54%
2020	Escherichia coli	30		17.54%		Acinetobacter baumannii	47	14.46%	Pseudomonas aeruginosa	3	27.27%		Escherichia coli	25	10.92%
	Staphylococcus aureus	16		9.36%		Staphylococcus aureus	21	6.46%	Escherichia coli	2	18.18%		Pseudomonas aeruginosa	22	9.61%
	Acinetobacter baumanni	15		8.77%		Pseudomonas aeruginosa	16	4.92%	Acinetobacter baumannii	1	9.09%		Candida albicans	17	7.42%
	Klebsiella pneumoniae	7		4.09%		Klebsiella pneumoniae	15	4.62%	Candida albicans	1	9.09%		Candida Tropicales	8	3.49%
	Enterococcus faecalis	4		2.34%		Candida albicans	5	1.54%	Candida parapsilosis	1	9.09%		Enterococcus faecalis	7	3.06%
2021	Escherichia coli	27		19.57%		Acinetobacter baumannii	82	24.33%	Acinetobacter baumannii	3	20.00%		Pseudomonas aeruginosa	37	13.05%
	Acinetobacter baumannii	19		13.77%		Pseudomonas aeruginosa	37	10.98%	Klebsiella pneumoniae	2	13.33%		Escherichia Coli	36	13.14%
	Staphylococcus aureus	10		7.25%		Klebsiella pneumoniae	29	8.61%	Pseudomonas aeruginosa	2	13.33%		Candida tropicalis	25	9.12%
	Klebsiella pneumoniae	9		6.52%		Staphylococcus aureus	22	6.53%	Staphylococcus epidermidis	2	13.33%		Candida albicans	20	7.30%
	Staphylococcus epidermidis	7		5.07%		Escherichia Coli	13	3.86%	Candida albicans	1	6.67%		Klebsiella pneumoniae	12	4.38%
2022	Escherichia coli	24		17.52%		Acinetobacter baumannii	77	21.63%	Achromobacter denitrificans	9	17.31%		Escherichia Coli	56	16.47%
	Staphylococcus aureus	14		10.22%		Pseudomonas aeruginosa	48	13.48%	Staphylococcus epidermidis	8	15.38%		Candida albicans	40	11.76%
	Acinetobacter baumannii	10		7.30%		Klebsiella pneumoniae	39	10.96%	Pseudomonas aeruginosa	7	13.46%		Pseudomonas aeruginosa	26	7.65%
	Enterococcus faecalis	5		3.65%		Staphylococcus aureus	31	8.71%	Acinetobacter baumanni	6	11.54%		Candida Tropicales	21	6.18%
	Staphylococcus epidermidis	5		3.65%		Escherichia coli	19	5.34%	Staphylococcus hominnis	5	9.62%		Candida glabrata	13	3.82%
2023	Escherichia coli	15		15.00%		Acinetobacter baumannii	47	20.43%	Staphylococcus epidermidis	6	2.61%		Escherichia coli	15	15.46%
	Staphylococcus aureus	8		8.00%		Pseudomonas aeruginosa	18	7.83%	Staphylococcus aureus	2	0.87%		Candida albicans	9	9.28%
	Acinetobacter baumannii	5		5.00%		Staphylococcus aureus	14	6.09%	Candida albicans	1	0.43%		Pseudomonas aeruginosa	6	6.19%
	Pseudomonas aeruginosa	5		5.00%		Klebsiella pneumoniae	12	5.22%	Corynebacterium spp.	1	0.43%		Candida tropicalis	4	4.12%
	Enterobacter cloacae	4		4.00%		Escherichia coli	11	4.78%	Klebsiella pneumoniae	1	0.43%		Candida glabrata	3	3.09%

The percentage of resistance was obtained from the total number of isolated microorganisms. Table 7 shows the percentage of antibiotic resistance for each of the study years.

**Table 7:** Total antibiotic resistance per year

Antibiotic		Gentamicina		Vancomicina		Ampicilina		Amoxicilina Ac. Clavulanico		Ceftriaxona		Eritromicina		Ciprofloxacino		Levofloxacino		Moxifloxacino	
Year	N	n (%)		n (%)		n (%)		n (%)		n (%)		n (%)		n (%)		n (%)		n	
2019	1015	440 (4%3)		4 (0.4%)		362 (36%)		1 (0.1%)		413 (41%)		148 (15%)		595 (59%)		134 (13%)		107	
2020	652	233 (36%)		12 (2%)		69 (11%)		30 (5%)		228 (35%)		92 (14%)		351 (54%)		90 (14%)		56	
2021	919	320 (35%)		15 (2%)		75 (8%)		130 (14%)		334 (36%)		128 (14%)		496 (54%)		111 (12%)		75	
2022	951	265 (28%)		7 (0.7%)		101 (11%)		141 (15%)		426 (45%)		127 (13%)		482 (51%)		108 (11%)		75	
2023	399	70 (18%)		5 (1%)		28 (7%)		29 (7%)		80 (20%)		25 (6%)		101 (23%)		22 (6%)		15	
Antibiotic		Doxiciclina		Tigeciclina		Linezolid		TMP/SMX		Amikacina		Piperacilina Tazobactam		Cefalotina		Cefazolina		Cefepime	
Year	N	n (%)		n (%)		n (%)		n (%)		n (%)		n (%)		n (%)		n (%)		n	
2019	1015	18 (2%)		48 (5%)		4 (0.4%)		453 (45%)		100 (10%)		87 (9%)		403 (40%)		154 (15%)		447	
2020	652	15 (2%)		67 (10%)		8 (1%)		95 (15%)		68 (10%)		131 (20%)		90 (14%)		154 (24%)		259	
2021	919	18 (2%)		99 (11%)		13 (1%)		137 (15%)		92 (10%)		211 (23%)		71 (8%)		9 (1%)		377	
2022	951	20 (2%)		102 (11%)		4 (0.4%)		177 (19%)		84 (9%)		181 (19%)		125 (13%)		24 (3%)		344	
2023	399	3 (0.8%)		15 (4%)		0 (0%)		23 (6%)		15 (4%)		47 (12%)		24 (6%)		0 (0%)		80	
Antibiotic		Cefotaxima		Ceftacidime		Colistina		Norfloxacino		Ertapenem		Imipenem		Meropenem		Nitrofurantoina		Global resistance (%)	
Year	N	n (%)		n (%)		n (%)		n (%)		n (%)		n (%)		n (%)		n (%)		%	
2019	1015	353 (35%)		312 (31%)		3 (0.3%)		165 (12%)		18 (2%)		12 (1%)		241 (24%)		136 (13%)		19.55%	
2020	652	117 (18%)		261 (40%)		9 (1%)		70 (11%)		22 (3%)		134 (21%)		165 (25%)		20 (3%)		16.79%	
2021	919	109 (12%)		375 (41%)		5 (0.5%)		80 (9%)		33 (4%)		198 (22%)		255 (28%)		18 (2%)		15.84%	
2022	951	119 (13%)		342 (36%)		8 (0.8%)		94 (10%)		5 (0.5%)		183 (19%)		244 (26%)		23 (2%)		15.41%	
2023	399	14 (4%)		70 (16%)		4 (1%)		15 (4%)		1 (0.3%)		31 (8%)		47 (12%)		6 (2%)		7.42%	

The analysis of antibiotic resistance by microorganisms does not show a trend regarding its increase or decrease. Table 8 shows the five most frequently isolated microorganisms per year and the percentage of resistance.

**Table 8:** Microbiological profile of the top five most common isolated bacteria by year

Year	Isolated microorganism		n	Gentamicina	Ampicilina	Amoxicilina Ac. Clavulanico	Ceftriaxona	Eritromicina	Ciprofloxacino	Levofloxacino	Moxifloxacino	Doxiciclina	Tigeciclina	TMP/SMX	Amikacina	Ampicilina Sulbactam	Piperacilina Tazobactam	Cefalotina	Cefazolina	Cefepime	Cefotaxima	Ceftacidime	Colistina	Norfloxacino	Ertapenem	Imipenem	Meropenem	Nitrofurantoina
																													2019	Escherichia coli		197	63.96%	89.34%	0.00%	75.13%	0.00%	82.23%	0.00%	0.00%	0.00%	0.00%	65.99%	6.60%	61.42%	5.58%	56.85%	22.84%	75.63%	52.79%	52.28%	0.00%	50.76%	3.55%	0.00%	1.02%	3.55%
																														Acinetobacter baumannii		173	71.68%	23.70%	0.00%	90.17%	0.00%	91.91%	0.58%	0.00%	0.00%	4.05%	87.28%	1.73%	84.97%	28.32%	66.47%	24.28%	90.75%	61.27%	61.85%	0.00%	0.58%	0.00%	4.62%	90.75%	23.12%
																														Pseudomonas aeuroginosa		125	48.00%	20.80%	0.00%	20.80%	0.00%	55.20%	0.00%	0.80%	0.00%	25.60%	21.60%	56.80%	20.80%	13.60%	73.60%	21.60%	49.60%	72.80%	40.00%	0.00%	36.00%	0.00%	2.40%	54.40%	22.40%
																														Klebsiella pneumoniae		91	41.76%	89.01%	0.00%	61.54%	0.00%	39.56%	0.00%	0.00%	0.00%	3.30%	48.35%	3.30%	49.45%	6.59%	39.56%	19.78%	61.54%	38.46%	40.66%	0.00%	7.69%	8.79%	1.10%	8.79%	26.37%
																														Staphylococcus aureus		80	1.25%	0.00%	0.00%	0.00%	45.00%	40.00%	38.75%	38.75%	0.00%	N/R	3.75%	N/R	0.00%	N/R	N/R	N/R	N/R	N/R	N/R	N/R	N/R	N/R	N/R	N/R	N/R
2020	Acinetobacter baumannii		116	71.55%	0.00%	1.72%	89.66%	0.00%	87.93%	0.00%	0.00%	0.00%	5.17%	5.17%	0.00%	85.34%	82.76%	4.31%	0.00%	88.79%	5.17%	87.07%	0.00%	0.00%	0.86%	82.76%	84.48%	0.00%
	Escherichia coli		99	48.48%	42.42%	13.13%	78.79%	0.00%	71.72%	0.00%	0.00%	0.00%	0.00%	32.32%	10.10%	62.63%	6.06%	40.40%	0.00%	79.80%	46.46%	76.77%	1.01%	37.37%	4.04%	0.00%	3.03%	2.02%
	Pseudomonas aeuroginosa		81	40.74%	1.23%	9.88%	0.00%	0.00%	56.79%	0.00%	0.00%	0.00%	60.49%	0.00%	56.79%	0.00%	22.22%	24.69%	0.00%	43.21%	34.57%	51.85%	0.00%	28.40%	0.00%	33.33%	60.49%	0.00%
	Klebsiella pneumoniae		52	32.69%	26.92%	5.77%	53.85%	0.00%	51.92%	0.00%	0.00%	0.00%	11.54%	17.31%	17.31%	51.92%	13.46%	21.15%	0.00%	53.85%	32.69%	55.77%	0.00%	11.54%	25.00%	11.54%	25.00%	15.38%
	Staphylococcus aureus		52	3.85%	0.00%	0.00%	0.00%	25.00%	32.69%	32.69%	30.77%	0.00%	N/R	9.62%	N/R	0.00%	N/R	N/R	N/R	N/R	N/R	N/R	N/R	N/R	N/R	N/R	N/R	N/R
2021	Acinetobacter baumannii		183	46.45%	0.00%	7.10%	89.62%	0.00%	91.80%	0.00%	0.00%	0.00%	4.37%	8.74%	0.55%	54.10%	85.79%	1.09%	2.19%	92.35%	7.10%	88.52%	0.00%	0.00%	0.55%	82.51%	90.71%	0.00%
	Escherichia coli		140	56.43%	35.00%	35.71%	77.14%	0.00%	79.29%	0.00%	1.43%	0.00%	0.00%	25.71%	9.29%	52.14%	9.29%	31.43%	0.00%	75.71%	27.14%	76.43%	0.71%	29.29%	7.14%	3.57%	4.29%	1.43%
	Pseudomonas aeuroginosa		120	44.17%	0.83%	36.67%	0.83%	0.00%	45.83%	0.00%	0.00%	0.00%	60.00%	0.00%	50.00%	0.00%	19.17%	1.67%	1.67%	40.00%	30.00%	42.50%	0.00%	21.67%	0.83%	25.00%	51.67%	0.83%
	Klebsiella pneumoniae		80	26.25%	22.50%	18.75%	57.50%	0.00%	42.50%	0.00%	0.00%	1.25%	10.00%	18.75%	16.25%	48.75%	11.25%	18.75%	2.50%	56.25%	16.25%	53.75%	5.00%	12.50%	18.75%	10.00%	18.75%	12.50%
	Staphylococcus aureus		53	3.77%	0.00%	0.00%	0.00%	28.30%	22.64%	22.64%	22.64%	0.00%	N/R	0.00%	N/R	0.00%	N/R	N/R	N/R	N/R	N/R	N/R	N/R	N/R	N/R	N/R	N/R	N/R
2022	Acinetobacter baumannii		153	42.48%	0.00%	5.88%	90.85%	0.00%	91.50%	0.00%	0.00%	1.31%	1.96%	15.69%	0.00%	56.86%	85.62%	5.88%	9.80%	87.58%	5.23%	81.05%	0.00%	0.00%	0.00%	73.86%	89.54%	0.00%
	Escherichia coli		141	44.68%	53.19%	52.48%	70.21%	0.00%	78.72%	0.00%	1.42%	0.00%	0.00%	46.81%	4.96%	50.35%	10.64%	41.13%	0.00%	70.92%	39.72%	68.09%	0.00%	39.72%	0.00%	2.84%	4.26%	4.96%
	Pseudomonas aeuroginosa		124	39.52%	0.81%	24.19%	86.29%	0.00%	50.00%	0.00%	0.00%	0.00%	69.35%	0.81%	45.97%	0.81%	4.84%	24.19%	4.03%	37.90%	22.58%	40.32%	0.81%	18.55%	0.00%	37.10%	52.42%	0.00%
	Klebsiella pneumoniae		71	25.35%	19.72%	16.90%	54.93%	0.00%	49.30%	0.00%	0.00%	0.00%	2.82%	9.86%	2.82%	54.93%	22.54%	12.68%	0.00%	52.11%	18.31%	54.93%	1.41%	7.04%	2.82%	15.49%	19.72%	5.63%
	Staphylococcus aureus		63	7.94%	0.00%	0.00%	0.00%	26.98%	28.57%	28.57%	25.40%	1.59%	N/R	0.00%	N/R	0.00%	N/R	N/R	N/R	N/R	N/R	N/R	N/R	N/R	N/R	N/R	N/R	N/R
2023	Acinetobacter baumannii		80	27.50%	6.25%	3.75%	36.25%	0.00%	42.50%	1.25%	0.00%	1.25%	0.00%	5.00%	5.00%	21.25%	37.50%	3.75%	0.00%	42.50%	2.50%	36.25%	0.00%	0.00%	0.00%	32.50%	41.25%	0.00%
	Escherichia coli		52	30.77%	25.00%	26.92%	38.46%	0.00%	46.15%	0.00%	0.00%	0.00%	0.00%	15.38%	0.00%	23.08%	9.62%	23.08%	0.00%	50.00%	9.62%	38.46%	1.92%	15.38%	1.92%	0.00%	1.92%	1.92%
	Pseudomonas aeuroginosa		42	14.29%	0.00%	11.90%	30.95%	0.00%	14.29%	0.00%	0.00%	0.00%	35.71%	0.00%	14.29%	0.00%	0.00%	11.90%	0.00%	11.90%	9.52%	11.90%	0.00%	9.52%	0.00%	4.76%	14.29%	0.00%EsZ
	Staphylococcus aureus		36	8.33%	0.00%	0.00%	0.00%	8.33%	8.33%	8.33%	11.11%	0.00%	N/R	0.00%	N/R	0.00%	N/R	N/R	N/R	N/R	N/R	N/R	N/R	N/R	N/R	N/R	N/R	N/R
	Staphylococcus epidermidis		30	10.00%	0.00%	0.00%	0.00%	36.67%	26.67%	30.00%	23.33%	3.33%	N/R	20.00%	N/R	0.00%	N/R	N/R	N/R	N/R	N/R	N/R	N/R	N/R	N/R	N/R	N/R	N/R
	Klebsiella pneumoniae		29	17.24%	20.69%	13.79%	31.03%	0.00%	34.48%	0.00%	0.00%	0.00%	0.00%	6.90%	6.90%	20.69%	17.24%	3.45%	0.00%	37.93%	0.00%	31.03%	6.90%	3.45%	0.00%	6.90%	17.24%	6.90%

## Discussion

The results show evidence of a decrease in the frequency of HAIs compared to pre-pandemic figures after post-pandemic implementation of Prevention and Control Infection strategies suggested by the WHO [10]. It is important to mention that the unit where the study was carried out is one of the largest in Mexico and that the results have the potential to be used as a national reference for public sector hospitals. The objective is not to compare the results obtained with those of other hospitals in Mexico and other places in the world, since each hospital may have various economic, organizational, cultural, political, and environmental factors that could make it unlikely to replicate the strategy that led us to not return to the figures from 2019 and previously regarding the number of HAIs.

Epidemiology and current panorama

During recent years, the pandemic derived from SARS-CoV-2, in addition to other epidemiological problems, has highlighted the potential damage that HAIs can cause to patients and healthcare personnel. As a result of these events, WHO published the First World Report on Infection Prevention and Control (IPC) in 2022 [10]. Previous reports from the same WHO and the Pan American Health Organization (PAHO) in 2015 indicated that globally, HAIs already represented a public health problem due to their social and economic significance, adding to the potential impact on patient safety. The report provides information regarding data from 2015 in which it was estimated that HAIs increased to an average of 7.5 days of hospital stay, with a specific mortality of 6.9% [11]. The problem is not minor, since it is estimated that in Europe alone, 3.2 million people acquire HAIs. In the United States, the reported rate is one for every 136 hospitalized patients. There is a difference in the frequency of HAIs between developed and developing countries (12% vs. 25%) [11]. The above also generates an increase in the mortality rate, resistance to antibiotics, and excessive costs to healthcare systems. In 2017, the WHO estimated that in Mexico there were approximately 450,000 cases of HAIs and a mortality rate of 32 deaths per 100,000 inhabitants [11]. The bulletin, published by CONAMED-PAHO in 2018, states that the frequency of HAIs in our country is approximately 21%, which increases to 23% when patients are treated in intensive care units [11]. The report also describes an analysis that covers from 2005 to 2015, with an average of HAIs in Mexico of 4.7 per 100 hospital discharges (61,969 HAIs in 2015), which in 2010 presented the highest rate of 8.2 per 100 hospital discharges. It is important to note that this report only includes one IMSS medical unit. The data that IMSS can provide is relevant, since at the end of 2021 the number of beneficiaries in the ordinary regime was reported at 71.6 million plus 11.6 million beneficiaries of IMSS-BIENESTAR [12].

Given this context, it can be assumed that IMSS practically serves more than half of the population of Mexico. This, when transformed into medical care, hospital discharges, and other statistical data, makes us think about the complexity and calculation of the rate of HAIs in the institution. As in many places in the world, the issue of infection control and prevention also became ongoing in our country. Today, however, it is possible to verify the frequency of HAIs in each of the IMSS units, yet the data, obtained from one of the largest units with more than 17 million beneficiaries, shows that the number of HAIs is underrated. The main cause of the figures is the lack of timely notification or no notification whatsoever of HAI cases. The Dirección General de Epidemiología (The Mexican General Directorate of Epidemiology) has issued recommendations for several years, which were strengthened in 2019 and 2020 [13]. They encourage the implementation of action packages and strategies that promote active surveillance daily involving personnel who care for the patient directly by employing a multidisciplinary strategy, assisted by the Epidemiological Surveillance Unit (ESU), to achieve satisfactory compliance with timely notification [14,15].

Antibiotic resistance

Due to a notable increase in recent years, antibiotic resistance is one of the greatest concerns of the WHO in our global environment [16]. One of the main causes of resistance is the discretionary use of medications without adherence to local and regional empirical guidelines. The ES of HAIs functions as an instrument to measure morbidity and mortality. Its usefulness is reflected in the success of the implementation of IPC packages [17].

Antibiotic resistance persists as a threat to public health. In 2019 alone, the CDC reported a global level of 5,000,000 HAIs and 1.27 million deaths associated with these types of infections around the world. In the United States, approximately 2.8 million antimicrobial-resistant infections are reported per year and are directly related to 35,000 deaths in 2019 [18,19]. WHO, together with the governments of several countries associated with the United Nations (UN), has closed ranks to reduce and prevent antibiotic resistance. In 2019, during the World Economic Forum in Switzerland, one of the most important topics discussed was taking action to efficiently counter the rapid and massive spread of infectious diseases, including HAIs. This accounts for 700,000 deaths per year worldwide due to antibiotic-resistant bacteria. The authors estimate that by 2050, 10,000,000 deaths per year are expected [19]. The ability of bacteria to evolve and acquire resistance to antibiotics has been an ongoing topic ever since antibiotics started being widely used. However, in recent years, knowledge about the molecular mechanisms of the development of resistance has been enriched, thereby promoting prevention strategies, control of infections, and rational use of antibiotics. These advances are derived from genomic, proteomics, and structural biology technology and have been a fundamental part of establishing and updating new ways to detect resistance, manage complex cases, and the appropriate use of new antibiotics [20].

The HAIs problem is not new. What was previously called nosocomial infections have been associated with the history of medical care for many years. In recent years, it has become more visible since we are currently more vulnerable to acquiring them. The above is secondary to longer life expectancy, chronic diseases, and advanced surgery through the use of prostheses or other devices that facilitate the generation of an HAI.

The recommendations are relatively the same. The Global Report on IPC [10] contains IPC packages very similar to those established in national regulations. We must also take into consideration that each hospital has different needs and populations with different vulnerabilities, which makes it difficult for a very general strategy to work everywhere.

The most important recommendation is to carry out an adequate health (or situational) diagnosis in which the conditions of the hospital can be evaluated. With this, it is also possible to detect the strengths and weaknesses of not only the hospital in general but also identify areas of opportunity and specific errors in distinct physical areas and groups of agents.

The second recommendation is the culture of ongoing education. It is necessary to maintain current courses regarding HAIs for all health personnel that emphasize both the concepts and action packages. Statistical data such as fatality, the impact on the quality of life of those who suffer from it, and the costs to the health sector must be shared. Ongoing training of healthcare personnel must be reestablished as one of the indicators for the adequate implementation of IPC packages.

Implement the necessary resources to strengthen epidemiological surveillance of HAIs. A proactive and multidisciplinary exercise to obtain more precise statistics and indicators that can project a clearer public health context is required. Sharing successful experiences in specialized forums on the subject and thus disseminating strategies to achieve more efficient notification and prevention and control measures for HAIs is essential [21].

As a global public health problem, HAIs have generated human losses and great costs to healthcare systems. For many years, strategies have been in force, but to a certain extent, static, since the feedback from the results obtained in recent years generates a repetition of the same recommendations. Implementing action packages requires preparing an entire field or a fairly complex organizational structure such as the IMSS hospital network, which is one of the largest in the world. Action packages must stop being universal, and the strategy must focus on establishing work plans based on the needs of each second- and third-level hospital.

The present work has some limitations, and the most important is that the research was performed in just one medical facility. However, the experience acquired in the field of action for the prevention and control of HAIs leaves us with several unknowns that are still to be resolved. It is difficult to explain why healthcare personnel do not report infections. Despite all of the efforts to discover the answer, it is difficult to obtain more than silence from the actors. In this particular case, when the first strategy was implemented in which service medical chiefs were notified about the presence of an infection in their patients, all the facilities were provided with this information to issue the required notification, which would have taken no more than five minutes. The service that presents the most infections has an average of 25 per month, which does not imply an excessive bureaucratic burden. Even so, services with four infections per month did not report them, despite having received notice to do so. However, in spite of these problems, we managed to make a noticeable gain from an average notification of 4% to 50% in just three months.

The data obtained from the 50% notification is not sufficient to be able to compile accurate statistics and to advance a work plan for future months. With incomplete data, one can only speculate about what may happen, and therefore, it is not possible to solve a problem that becomes increasingly serious with each passing day. In fact, for the World Health Organization, the development of multidrug-resistant organisms is a matter of concern and is closely linked to HAIs [10]. The increase in these types of microorganisms worldwide is one of the most important variables regarding the vulnerability of healthcare systems. With time and the same practices, it is not impossible to reach a total lack of control and be left completely without ways to contain an outbreak due to a bacterium resistant to all antibiotics. Nor is it about creating new antibiotics with a greater spectrum of action, since if we follow the same course of action, it will end up being useless against bacteria.

It is urgent to establish a model that educates, updates, and raises awareness about the vulnerability of human life to nature. The quality of life of future generations depends largely on the actions and decisions we fear today. Radically speaking, the continuity of the human species may also be in danger from the very microorganisms created by nature, which were enhanced by their own victims.

## Conclusions

HAIs are an ever-increasing global public health concern. Prevalence rates are increasing faster, especially in developing countries with poor resources and infrastructure, which warrants the correct application of control and prevention programs as outlined by the WHO. In the same way, public policy transfer regarding prevention should be particularized and based on a situational diagnosis. It’s not possible to apply the same strategies since each country has different and multiple panoramas to its approach. A microbiological profile is one of the most important steps for the initial diagnosis for each medical facility. Only with profound knowledge, coupled with resistance to antibiotics, can a better decision be made.

Regarding public policy and the organizational panorama in public health institutions, here are some lessons that have been learned from the present research. Public organizations that provide healthcare services are abstract entities. Their structure and functioning depend on the agents that comprise it, who are beings with mental sense and rational thought. Given the problem, it is necessary to have a strategic plan prior to implementation. This plan must allow corrections to be made along the way and, above all, to analyze any possible obstacles during planning. Preparing the field or arena is of vital importance. The best strategy is to provide awareness and training and obtain feedback from results. During planning, it is necessary to have the support of the authorities and to involve the highest-level actors in the organization so that there is a hierarchical order to achieve favorable results. Despite any disagreements on the part of the operatives and the image of correction that this may convey, we must remember that we are public servants and that we work in accordance with institutional and federal regulations. We must establish links with our peers in other health institutions who share the same characteristics to learn about their strategies and results. The transfer of public policies has shown exceptional results worldwide. An institutional improvement plan could have similar results. Leadership and the ability to present the magnitude of a problem are matters of great importance. Knowledge of the problems, safety, and clear accountability strengthen trust and generate the formation of social networks and the critical masses of public servants in general, which in turn improves the quality of the services provided by institutions.
